# Studies on the Requirement of Transthyretin Protein (BxTTR-52) for the Suppression of Host Innate Immunity in *Bursaphelenchus xylophilus*

**DOI:** 10.3390/ijms232315058

**Published:** 2022-12-01

**Authors:** Tong-Yue Wen, Yan Zhang, Xiao-Qin Wu, Jian-Ren Ye, Yi-Jun Qiu, Lin Rui

**Affiliations:** 1 Co-Innovation Center for Sustainable Forestry in Southern China, College of Forestry, Nanjing Forestry University, Nanjing 210037, China; 2 Jiangsu Key Laboratory for Prevention and Management of Invasive Species, Nanjing Forestry University, Nanjing 210037, China

**Keywords:** *Bursaphelenchus xylophilus*, *Pinus thunbergii*, transthyretin, pathogenic mechanism, suppress immune response

## Abstract

The pinewood nematode, *Bursaphelenchus xylophilus*, has been determined as one of the world’s top ten plant-parasitic nematodes. It causes pine wilt, a progressive disease that affects the economy and ecologically sustainable development in East Asia. *B. xylophilus* secretes pathogenic proteins into host plant tissues to promote infection. However, little is known about the interaction between *B. xylophilus* and pines. Previous studies reported transthyretin proteins in some species and their strong correlation with immune evasion, which has also been poorly studied in *B. xylophilus*. In this study, we cloned and functionally validated the *B. xylophilus* pathogenic protein BxTTR-52, containing a transthyretin domain. An in situ hybridization assay demonstrated that BxTTR-52 was expressed mainly in the esophageal glands of *B. xylophilus*. Confocal microscopy revealed that BxTTR-52-RFP localized to the nucleus, cytoplasm, and plasma membrane. BxTTR-52 recombinant proteins produced by *Escherichia coli* could be suppressed by hydrogen peroxide and antioxidant enzymes in pines. Moreover, silencing BxTTR-52 significantly attenuated the morbidity of *Pinus thunbergii* infected with *B. xylophilus*. It also suppressed the expression of pathogenesis-related genes in *P. thunbergii*. These results suggest that BxTTR-52 suppresses the plant immune response in the host pines and might contribute to the pathogenicity of *B. xylophilus* in the early infection stages.

## 1. Introduction

Pinewood nematode (PWN; *Bursaphelenchus xylophilus*) is a pathogen that causes pine wilt disease (PWD), which is one of the most devastating forest diseases in the world [1]. PWD has spread throughout the world via trade activities and resulted in enormous economic losses and ecological problems in many countries, such as Japan, China, Korea, and Portugal [2,3]. The pathogenic mechanism of PWD is complicated and involves many pathogenic factors including host pines, nematodes, vector insects, microorganisms, environmental factors, and other aspects. Meanwhile, *B. xylophilus* has two different life cycles, one a propagative form under suitable conditions, and the other a dispersal form [4]. To reduce loss and prevent infection spread, we need to understand the pathogenesis of PWN and how it interacts with pines.

The genomic data of *B. xylophilus* indicate that this nematode has many pathogenesis-related genes. Some plant cell-wall-degrading enzymes (CWDEs) such as carbohydrate-active enzymes (CAZymes) and expansin-like proteins may modify plant cell walls detected in *B. xylophilus* [5,6]. They have roles as pathogenicity determinants, such as glycoside hydrolase family 45 cellulases, pectate lyases, and b-1,3-endoglucanases [7,8]. Peptidases (proteases) are involved in a broad range of pathological processes that catalyze the cleavage of peptide bonds within proteins to establish parasitism [9]. Additionally, *B. xylophilus* must cope with a wide range of secondary metabolites of its host, including terpenoids and cyclic aromatic compounds [10,11]. It seems likely that *B. xylophilus* has a larger number of genes involved in regulating the detoxification process. *B. xylophilus* evolves different pathogenic genes to adopt its complex ecology and promote infection.

Transthyretin (TTR) proteins play multi-functional roles in nematodes that have been involved in the host–parasite interactions [12,13,14]. TTR is a transport protein in extracellular fluids, where it distributes the two thyroid hormones, 3,5,3′-triiodo-L-thyronine (T3) and 3,5,3′,5′-tetraiodo-L-thyronine (thyroxine, T4), as well as vitamin A in complex with retinol-binding proteins [15,16]. It has been proposed that TTR proteins play various roles in regulating apoptosis and modulating host immune responses in a range of organisms. For example, *Caenorhabditis elegans* transthyretin-like protein TTR-52 mediates the recognition of apoptotic cells by the CED-1 phagocyte receptor [17]. A transthyretin-like protein MjTTL5 from *Meloidogyne javanica* suppresses oxidative response through the cunning exploitation of the host’s ROS-scavenging system [18]. However, transthyretin-like protein has rarely been reported in *B. xylophilus*.

In order to successfully infect plants, pathogens and parasites must subvert plants’ immunity [19]. Among them, reactive oxygen species (ROS) play a major role in pathogen–plant interactions [20]. The major forms of ROS are singlet oxygen (O_2_), superoxide anion (O_2_^−^), hydrogen peroxide (H_2_O_2_), and hydroxyl radical (HO) in plants [21]. ROS are mainly involved in defense reactions, which function as diffusible second messengers to induce several resistance responses including compounds of pathogenesis-related proteins and programmed cell death in neighboring cells [22]. The main locations of ROS synthesis include mitochondria, plasma membranes, peroxisomes, endoplasm, and cell walls [23,24,25]. Accumulating evidence suggests that *B. xylophilus* can interfere with the ROS accumulation of pines, which is highly related to their pathogenicity [26].

In the present study, the spatial location and subcellular localization of BxTTR-52 were confirmed by in situ hybridization and transient expression assay. RNA interference assay revealed the function and influence of BxTTR-52 on host defense responses. This study sheds new light on the interaction between *B. xylophilus* and pines.

## 2. Results

### 2.1. BxTTR-52 Was Predominantly Expressed in Esophageal Glands

**Figure 1:**
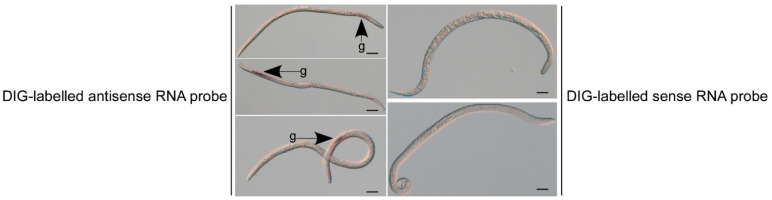
Localization of *BxTTR-52* in the dorsal glands by in situ hybridization. The scale bars = 20 µm.

The gene (BXY_0198900) was identified from the transcriptome of *B. xylophilus*, which was predicted to contain a transthyretin domain and is denoted as BxTTR-52 for further study. As most known nematode effectors are synthesized within the specialized secretory gland cells, we used an in situ hybridization assay with a digoxigenin (DIG)-labeled antisense probe to confirm the spatial expression pattern of BxTTR-52 in nematode tissues. An Open Reading Frame (ORF) of *BxTTR-52* was used to synthesize sense and antisense digoxigenin-labeled probes employing T7 RNA polymerase. After fixation, hybridization, and staining, a strong hybridization signal was observed in esophageal glands hybridized with antisense probes, with little background on *B. xylophilus* hybridized with sense probes (Figure 1), indicating that BxTTR-52 is a secretory protein in the nematode–pine interplay.

### 2.2. BxTTR-52 Localized on the Cytoplasm and Nucleus of Plant Cells

**Figure 2:**
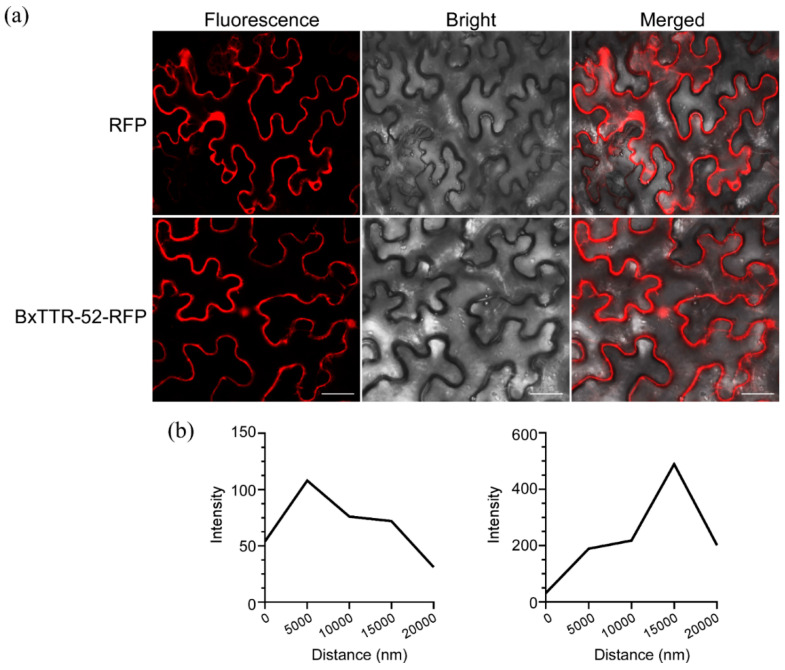
BxTTR-52 localized on the cytoplasm and nucleus of plant cells. (**a**) Subcellular localization of BxTTR-52 in *Nicotiana benthamiana*. The scale bar represents 50 μm. (**b**) The RFP intensity was delineated from the cytoplasmic and nuclear areas (**left** to **right**).

When effector proteins are secreted, they function either in the apoplastic spaces or inside host cells. The localization of pathogen effectors inside the plant cell gives an indication of their mode of action. To better understand the function of BxTTR-52 in plants, the coding sequence of BxTTR-52 was ligated into a plasmid pBINRFP and we analyzed the subcellular localization of functional BxTTR-52-RFP by a transient expression system using *N. benthamiana*. The confocal microscopy revealed that BxTTR-52-RFP mainly localized to the cytoplasm and plasma membrane. Our results showed that BxTTR-52 is weakly expressed in the nucleus (Figure 2a). The fluorescence intensity also demonstrated a reduction in the expression of the nucleus (Figure 2b), suggesting that BxTTR-52 is secreted into the intracellular site in plant cells.

### 2.3. BxTTR-52 Suppressed the Accumulation of ROS in Host Pines

**Figure 3:**
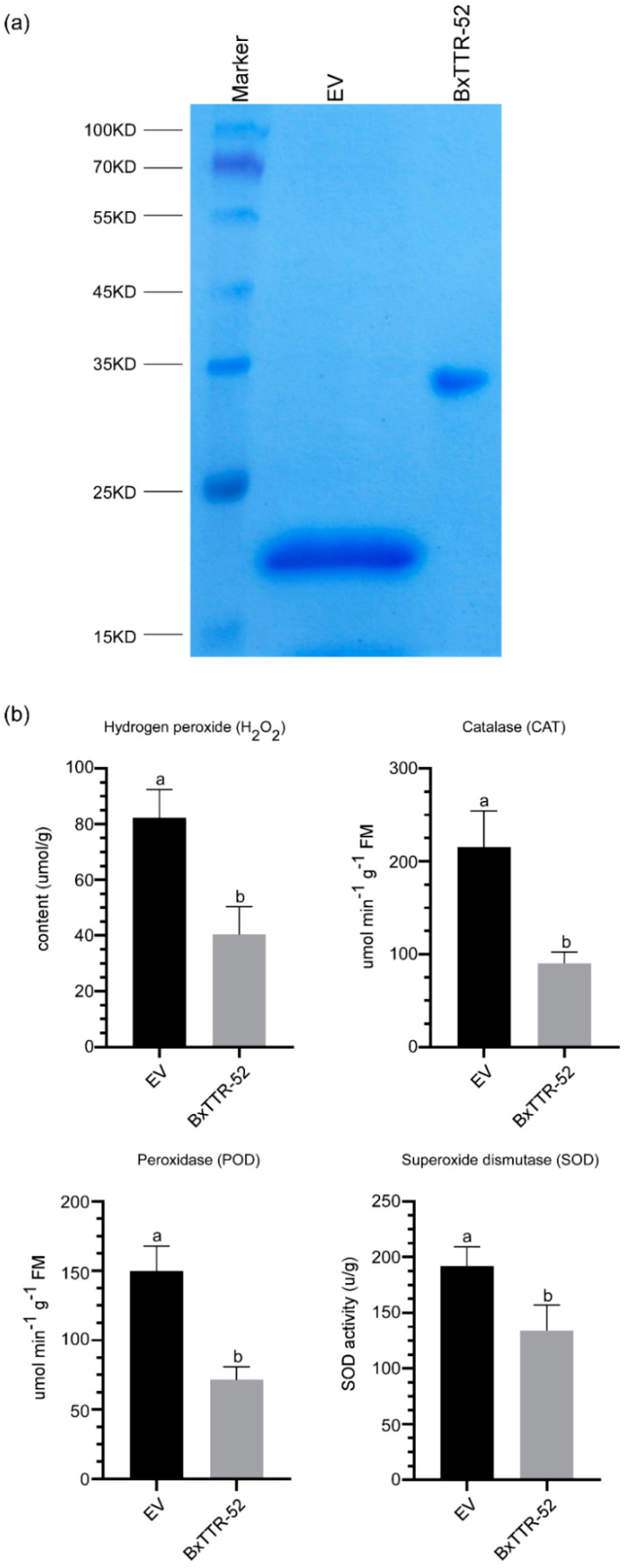
BxTTR-52 suppressed the accumulation of ROS and antioxidant enzymes. (**a**) SDS-polyacrylamide gel electrophoresis (SDS-PAGE) verification of the BxTTR-52 protein. (**b**) The content of ROS and antioxidant enzymes. Values represent the means *±* SD of three independent biological samples. Different letters on top of the bars indicate statistically significant differences (*p* < 0.05, *t*-test) as measured by Duncan’s multiple range test.

Due to comparable genetic tools are still not available in *P. thunbergii*, we attempted to purify the BxTTR-52 protein to explore its role in plant immunity. We succeeded in obtaining the recombinant proteins BxTTR-52rec produced in *Escherichia coli*. Meanwhile, BxTTR-52rec and EV were assessed by SDS polyacrylamide gel electrophoresis (SDS-PAGE) analysis to confirm successful purification (Figure 3a). It has become accepted that H_2_O_2_ and antioxidant enzymes play important roles in disease resistance. To further confirm the role of BxTTR-52 in the regulation of pines’ immunity, we injected purified BxTTR-52 protein into the stem of *P. thunbergii*, and *B. xylophilus* was inoculated 2 h later. EV was used as a negative control. The results showed that compared with the negative control, the accumulation of H_2_O_2_, catalase (CAT), peroxidase (POD), and superoxide dismutase (SOD) decreased in pines inoculated with purified BxTTR-52rec (Figure 3b), suggesting that BxTTR-52 might inhibit the host’s immunity during the *B. xylophilus* infection stage.

### 2.4. BxTTR-52 Did Not Affect B. xylophilus Feeding and Reproduction

**Figure 4:**
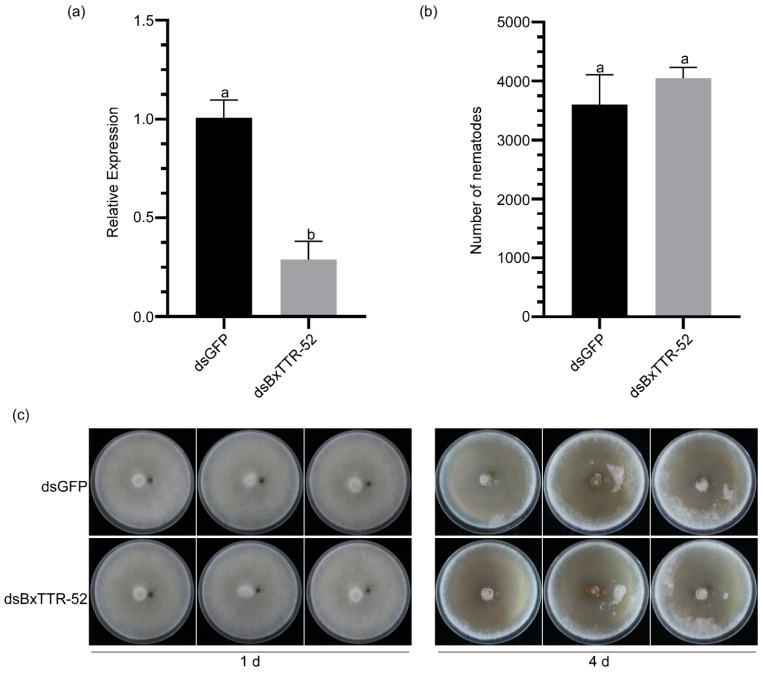
The effect of BxTTR-52 silencing on the reproduction and feeding rate of *Bursaphelenchus xylophilu*s. (**a**) The silencing efficiency of BxTTR-52 in *B. xylophilus*. (**b**) The number of nematodes on *Botrytis cinerea* over 4 days. (**c**) The propagating quantity of *B. xylophilus* cultured on *B. cinerea*. Values represent the means *±* SD of three independent biological samples. Different letters on top of the bars indicate statistically significant differences (*p* < 0.05, *t*-test) as measured by Duncan’s multiple range test.

To further confirm the effect of BxTTR-52 on *B. xylophilus* infection, we performed an RNA interference (RNAi) assay by soaking the nematodes in BxTTR-52 or GFP double-stranded RNA (siRNA). The result of real-time quantitative PCR (qRT-PCR) showed that the expression level of *BxTTR-52* in nematodes was decreased to 0.32, meaning that the RNAi assay was successful (Figure 4a). The reproductive trait of *B. xylophilus* is a vital factor for their pathogenicity. Hence, we inoculated the nematodes into PDA plates with *B. cinerea* to determine the effect of *BxTTR-52* on *B. xylophilus* reproduction. The feeding rate and reproduction of *B. xylophilus* were calculated after inoculation into *B. cinerea* for 4 d. The numbers of *BxTTR-52* siRNA-treated nematodes were a little higher than *GFP* siRNA-treated nematodes, but the difference was not significant (Figure 4b). Moreover, there was no obvious difference in the feeding rate of *BxTTR-52* siRNA-treated nematodes and *GFP* siRNA-treated nematodes (Figure 4c). The similar results obtained from the three treatments showed that *BxTTR-52* had little effect on the reproduction of *B. xylophilus*.

### 2.5. BxTTR-52 Contributed to Virulence during B. xylophilus Infection

**Figure 5:**
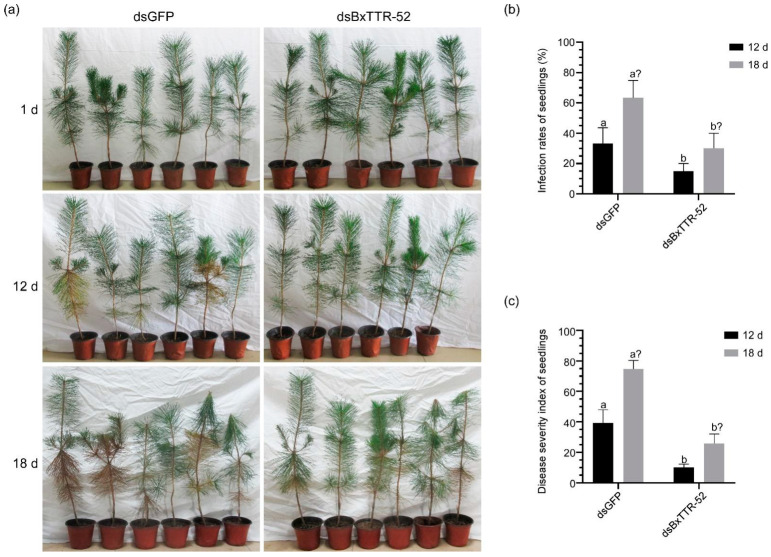
BxTTR-52 contributes to *Bursaphelenchus xylophilu*s virulence. (**a**) Inoculation assay of pine seedlings. Based on the color of the needles, the morbidity degree of the *Pinus thunbergii* seedlings was different. The seedlings inoculated with *dsGFP*-treated nematodes were the negative controls. (**b**) The infection rates of *P. thunbergii* seedlings under three different treatments. (**c**) The disease severity index of *P. thunbergii* seedlings under three different treatments. Values represent the means *±* SD of three independent biological samples. Different letters on top of the bars indicate statistically significant differences (*p* < 0.05, *t*-test) as measured by Duncan’s multiple range test.

We further explored whether BxTTR-52 affected the pathogenicity of *B. xylophilus* by the inoculation of RNAi-treated nematodes. For the infection assay, each 3-year-old *P. thunbergii* seedling was inoculated with *B. xylophilus* and treated with BxTTR-52 siRNA and GFP siRNA. We recorded the symptoms of pine seedlings inoculated with treated nematodes at various infection stages. The results showed that most seedlings infected with dsBxTTR-52-treated nematodes remained healthy and only one seedling turned slightly yellow, while *P. thunbergii* seedlings infected with dsGFP-treated nematodes exhibited distinct yellow needles at 12 days postinoculation (dpi). Until 18 dpi, all *P. thunbergii* seedlings treated with RNAi nematodes displayed symptoms, but *P. thunbergii* seedlings treated with dsBxTTR-52 nematodes had less disease (Figure 5a). The infection rate and disease severity index of the *P. thunbergii* seedlings inoculated with the nematodes treated with dsBxTTR-52 were obviously lower than those inoculated with the nematodes treated with dsGFP (Figure 5b), indicating that BxTTR-52 contributed to the virulence of *B. xylophilus* at the early infection stage.

### 2.6. The Expression of Defense-Related Genes Was Reduced in Host Pines Inoculated with RNAi Nematodes

**Figure 6:**
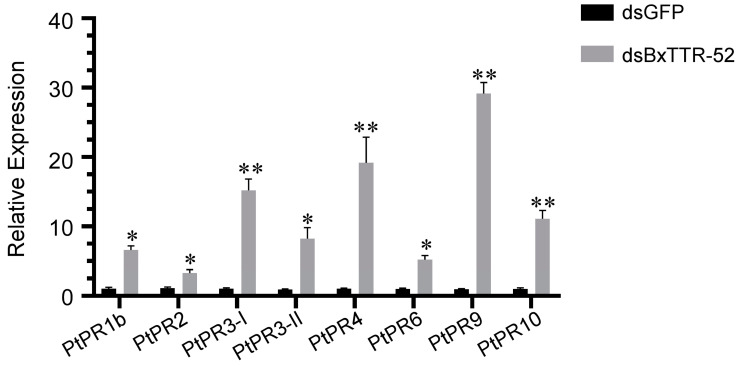
The relative expression of pathogenesis-related genes in *Pinus thunbergii* infected with *BxTTR-52* siRNA-treated nematodes. We selected stems ~2 cm in length to extract RNA at 12 h postinoculation. Values represent the means *±* SD of three independent biological samples. * indicated statistically significant differences (*p* < 0.05, *t*-test) as measured by Duncan’s multiple range test. ** indicated statistically significant differences (*p* < 0.01, *t*-test) as measured by Duncan’s multiple range test.

Pathogenesis-related (PR) proteins were one of the protective compounds in plants to resist the invasion of pathogens. In order to investigate whether BxTTR-52 influenced pine defense responses, *P. thunbergii* seedlings were inoculated with dsBxTTR-52-treated or dsGFP-treated *B. xylophilus*. We analyzed the expression of PR genes in pines after *B. xylophilus* inoculation using RT-qPCR. The results demonstrated that the expression level of most PR genes was distinctly upregulated in *P. thunbergii* infected with dsBxTTR-52-treated nematodes (Figure 6). Among them, the relative expression level of PtPR9 was 28.276, significantly higher than other treatments, implying that BxTTR-52 indeed influenced the defense responses of *P. thunbergii*.

## 3. Discussion

*B. xylophilus* is an essential threat to forest ecosystems worldwide. It is an unusual parasite, which represents the independent origin of plant parasitism nematode [27,28]. Until now, its pathogenic mechanism remains unclear. With the publication of the *B. xylophilus* genome, the transcriptome and secretome had been analyzed, highlighting parasitism genes linked to key biological processes [29]. Moreover, like other pathogens, a large number of pathogenesis-related genes were involved in the interaction between nematodes and hosts during the *B. xylophilus* infection stage. For example, cell-wall-degrading enzymes played a key role in the evolution of *B. xylophilus* [30]. The expansin-like genes were likely involved in assisting nematodes’ migration through the pines [31]. In this study, we reported that a transthyretin protein, BxTTR-52, is mainly located in esophageal glands. The modifying effect of esophageal line secretions on plant cell metabolism and its contents is important for the pathogenicity of *B. xylophilus* [32]. BxTTR-52 is expressed in esophageal glands, implying that *B. xylophilus* delivers, it via the stylet and BxTTR-52 might participate in the interaction between *B. xylophilus* and *P. thunbergii*.

The plant–pathogen interactions are complex, in which the pathogens attempt to invade successfully and the plants need to protect themselves from this invasion [33]. As we know, the oxidative burst is one of the earliest responses of plants to pathogen invasion [21]. In general, ROS are produced in many parts of the cells, such as the plasma membrane and multiple organelles [34]. We found that BxTTR-52 is located in the cytoplasm of plants, which might be closely related to resistance to ROS. On the other hand, ROS also had a signaling function mediating defense gene activation [35]. In order to overcome the plant defense, it was essential for parasitic nematodes to neutralize ROS. In this study, BxTTR-52 could suppress the accumulation of pines’ H_2_O_2_ and antioxidant enzymes during the infection stage, implying that *B. xylophilus* guaranteed its own survival and facilitated its ongoing and persistent infestations by weakening the resistance of host pines.

RNA interference (RNAi) has been demonstrated to be a powerful investigative tool for the identification of gene function to help improve our understanding of plant parasitic nematodes [36]. It has been widely studied that the function of the TTR-52 protein as a bridging molecule mediates apoptotic cell engulfment [17]. We tried to investigate its function in the pathogenesis of *B. xylophilus*. Feeding and reproduction ability are important conditions for the pathogenesis of *B. xylophilus* [37]. Our results indicate that RNAi-mediated TTR-52 expression in *B. xylophilus* has no significant effect on their feeding reproduction. However, it is worth noting that the virulence of *B. xylophilus* was significantly reduced and the disease was delayed when BxTTR-52 was silenced by establishing double-stranded RNA. Consistent with the above results, most pathogenesis-related genes of pine seedlings inoculated with *dsBxTTR-52*-treated nematodes were up-regulated, which also demonstrated that BxTTR-52 could inhibit pines’ immunity. In this regard, the PR9 protein was shown to have peroxidase activity [38], and peroxidase has an important role in the fine defense response of plants, especially in maintaining cellular ROS levels [39]. The expression level of PtPR9 was significantly upregulated after infection with TTR-52-silenced *B. xylophilus*, suggesting that TTR-52 affects plant cellular ROS regulation. On the other hand, double-stranded RNA (siRNA) activated the homologous mRNAs to inhibit their translation and transcription to silence genes. When siRNAs were injected into the nematode’s body, certain target genes became inactive [40]. This might be an effective way to delay pine wilt disease by targeting the silencing of pathogenic genes in nematodes. Nevertheless, sufficient microinjection with siRNA was a major technical challenge, which might provide an idea for controlling PWNs. The results and data from this study provide a foundation for further investigation of B. xylophilus biology and the development of novel pathogen control strategies.

## 4. Materials and Methods

### 4.1. Nematode Culture and Plant Materials

*B. xylophilus* isolate AMA3 originated from infected *P. massoniana* in Anhui Province, China [41]. *B. xylophilus* was cultured on potato dextrose agar (PDA) plates covered with *Botrytis cinerea* mycelia at 25 °C for 7 days. Finally, *B. xylophilus* was collected using the Baermann funnel technique. *B. xylophilus* and *B. cinerea* were supplied by the Forest Protection Laboratory, Nanjing Forestry University. *N*. *benthamiana* was cultivated in a growth room for 5–6 weeks at 22 °C in 16 hr light/8 hr dark (16/8 LD) cycles. Three-year-old *P. thunbergii* seedlings were obtained from the experimental field of Nanjing Forestry University (Jurong Yaolingkou Forest Farm, Jiangsu, China) in October 2019 and were cultivated at temperatures ranging from 28 to 32 °C with relative humidity ranging from 65% to 75%. Each three-year-old *P. thunbergii* seedling was inoculated with approximately 2000 nematodes.

### 4.2. Plasmids

We used the homologous recombination method to construct plasmids. The total RNA was isolated from mixed-life-stage nematodes using the TRIzol reagent (Invitrogen, Carlsbad, CA, USA). The cDNA was synthesized using HiScript II Q RT SuperMix for qPCR (+gDNA wiper) (Vazyme Biotech Co., Ltd., Nanjing, China). The fragments for cloning were PCR-amplified using Phanta Max Super-Fidelity DNA Polymerase (Vazyme Biotech Co., Ltd., Nanjing, China); the corresponding primers are listed in the Supporting Information (Table S1). The gene BxTTR-52 was cloned from *B. xylophilus* isolate AMA3, based on transcriptomic data [42]. The fragment, including BxTTR-52, was cloned into the *Sma*I (New England Biolabs, Ipswich, MA, USA) restriction site of pBinRFP for subcellular localization by a ClonExpress II One Step Cloning Kit (Vazyme Biotech Co., Ltd., Nanjing, China). Likewise, pET-32a and BxTTR-52 (without signal peptide) fragments were digested by *EcoR*I (New England Biolabs) in the appropriate conditions for prokaryotic expression.

### 4.3. In Situ Hybridization

The coding region of BxTTR-52 was used as the template to synthesize the DIG-labeled sense RNA probe and antisense RNA probe using the DIG Northern Starter Kit (Roche Diagnostics, Mannheim, Germany). In situ hybridization (ISH) was performed according to the protocol of de Boer et al. The DIG-labeled sense RNA probe was used as a negative control. *B. xylophilus* underwent a series of treatments such as fixation, hybridization, and staining. Samples were observed and photographed with a Zeiss Axio Image M2 microscope (Zeiss, Oberkochen, Germany).

### 4.4. Confocal Microscopy

The coding region of BxTTR-52 was cloned into vector pBIN-RFP (red fluorescent protein). *Agrobacterium tumefaciens* strain GV3101 was transformed with recombinant plasmid and then used for the infiltration of the leaves of 4-week-old *N. benthamiana* plants. *A. tumefaciens* cells (OD_600_ ~ 0.4–0.6) were infiltrated in *N. benthamiana* leaves with needleless syringes. For fluorescence observations, patches of *N. benthamiana* leaves were cut after 36–48 hpi and used for confocal imaging on an LSM710 microscope (Zeiss, Oberkochen, Germany) with a 40× objective lens.

### 4.5. Prokaryotic Expression of the Recombinant BxTTR-52 Protein

The coding region of BxTTR-52 was inserted into pET32a, which contains a 6 × His tag. The construct was transformed into *Escherichia coli* JM109-competent cells. Individual colonies from the construct were tested by PCR for insertions, and the selected clones were verified by sequencing. The constructed plasmid was transformed into the *E. coli* strain BL21. Positive clones were grown in Luria–Bertani (LB) medium containing 100 µg/mL ampicillin. When the OD reached 0.6, Isopropyl-beta-D-thiogalactopyranoside (IPTG) was added into the LB medium to induce protein at 37 °C for 4 h. Then, the cultures were centrifuged at 8000 rpm for 10 min to procure the bacteria precipitate containing BxTTR-52 and EV, respectively. The precipitate was crushed and confirmed by SDS-PAGE. The purification of the recombinant protein from the supernatant was performed by affinity chromatography using Ni-NTA Superflow resin (Qiagen).

### 4.6. Determination of H_2_O_2_ Levels and Antioxidant Enzyme Activity in Pines

To analyze the role of BxTTR-52 in the regulation of pines’ immunity, the level of H_2_O_2_ and the activities of superoxide dismutase (SOD), catalase (CAT), and peroxidase (POD) were determined in the stem of *P. thunbergia*-injected purified BxTTR-52 protein and PWN inoculation was performed 2 h later. The stems were frozen in liquid nitrogen and ground to a fine powder using a mortar and pestle. The contents of H_2_O_2_, SOD, CAT, and POD were calculated based on previous studies [43]. The levels of H_2_O_2_ in leaves were determined based on the absorbances at 436 nm. The activities of SOD, CAT, and POD were determined based on the absorbances at 560, 240, and 470 nm, respectively.

### 4.7. In Vitro RNAi of BxTTR-52 Gene

BxTTR-52 siRNA and the control GFP siRNA were synthesized in vitro with specific primers using the in vitro Transcription T7 Kit (for siRNA Synthesis) (Takara, Japan) following the manufacturer’s instructions. Subsequently, approximately 10,000 *B. xylophilus* were soaked in 50 μL BxTTR-52 siRNA and GFP siRNA for 36–48 h in a shaking incubator at 20 °C with a rotation rate of 180 rpm in the dark. The nematodes from each treatment were thoroughly washed with ddH_2_O three times after soaking. Then, approximately 3000 nematodes from each treatment were used to extract RNA and synthesize cDNA. The cDNA was used for qRT-PCR to evaluate the efficiency of RNAi.

### 4.8. Reproduction Assay

Each *B. cinerea*-colonized PDA plate was inoculated with 40 individuals of the *B. xylophilus* after treatment with BxTTR-52 siRNA and GFP siRNA, respectively. The PDA plates were cultured in the dark at 25 °C for 4 days of inoculation. At the same time, each pine seedling was inoculated with 2000 mixed-stage nematodes after treatment with *BxTTR-52* siRNA and *GFP* siRNA, respectively, and these seedlings were grown in the phytotron until the control plants had withered entirely. Treatment with *GFP* siRNA was used as a control. Subsequently, the Baermann funnel method was used to collect all nematodes from the PDA plates. The number of nematodes was counted with an optical microscope (Leica DM500, Leica Microsystems, Heerbrugg, Switzerland). The two experiments above were both performed three times and each treatment had three replicates.

### 4.9. PWN Inoculation Assay

A sterile blade was used to cut a small wound deep into the xylem on three-year-old pine stems, and a cotton ball was inserted. The incision and cotton ball were then covered with a funnel-shaped parafilm. A 100 μL suspension (approximately 2000 mixed-stage nematodes) was pipetted into the xylem in each pine. The seedlings inoculated with *GFP* siRNA-treated nematodes were used as the negative control. According to the color of the needles, PWD symptoms were evaluated and categorized using five levels [44]: 0, all needles are green; I, a quarter of the needles have turned yellow; II, approximately half of the needles have turned yellow or brown; III, three-fourths of the needles have turned brown; and IV, the entire seedling has withered. The morbidity and morbidity degrees of pines were calculated by PWD symptoms as described previously [45].

### 4.10. Real-Time Quantitative PCR

Total *B. xylophilus* RNA was extracted with TRIzol (Invitrogen, California, USA). The total RNA of pine was extracted with an RNA Isolation Kit (TIANGEN, Beijing, China) according to the manufacturer’s protocol. Approximately 2 μg of RNA was used for cDNA synthesis using HiScript II 1st Strand cDNA Synthesis Kit (+gDNA wiper) (Vazyme Biotech Co., Ltd., Nanjing, China). With 1.0 μL of 1:5 diluted cDNA as the template, qRT-PCR was performed in a 20 μL reaction volume with ChamQ SYBR Color qPCR Master Mix (Vazyme Biotech Co., Ltd., Nanjing, China). The RT-qPCRs were performed on an ABI Prism 7500 PCR instrument under the following conditions: 95 °C for 2 min, 40 cycles at 95 °C for 30 s, and 60 °C for 30 s to calculate cycle threshold values, followed by a dissociation program of 95 °C for 15 s, 60 °C for 1 min, and 95 °C for 15 s to obtain melt curves. The relative expression values were determined using elongation factor-1 alpha as a reference gene and calculated with the formula 2^−ΔΔCt^. Gene expression levels were calculated on the basis of three technical replications.

## 5. Conclusions

Here, we identified and characterized the *B. xylophilus* transthyretin protein, BxTTR-52, which suppressed host immune responses and played an important role in the interaction between *B. xylophilus* and pines. Our study further explored the molecular mechanism of *B. xylophilus* causing PWD to better control this destructive pest.

## Data Availability

The data presented in this study are available on request from the corresponding author.

## Supplementary Materials

The supporting information can be downloaded at: https://www.mdpi.com/article/10.3390/ijms232315058/s1.
